# Tailoring Lipid-Based Drug Delivery Nanosystems by Synchrotron Small Angle X-ray Scattering

**DOI:** 10.3390/pharmaceutics14122704

**Published:** 2022-12-02

**Authors:** Barbara Sartori, Benedetta Marmiroli

**Affiliations:** Institute of Inorganic Chemistry, Graz University of Technology, Stremayrgasse 9/4, 8010 Graz, Austria

**Keywords:** lipid nanoparticles, drug delivery, SAXS, structural analysis, external stimuli response, in situ

## Abstract

Thanks to specific physico-chemical properties, drug delivery systems based on nanoparticles have proven to effectively transport delicate molecules for therapeutic purposes, protecting them from degradation, increasing their stability in the blood circulation and allowing to convey and release the transported substances in specific areas of the body. Nanoparticles obtained from biopolymers for applications in medicine and pharmaceutics have become particularly popular in recent years due to the enormous research effort in the field of vaccines to respond to the pandemic emergency. Among the various types of biopolymers used to produce nanoparticles for therapeutics, lipids have characteristics that make them biocompatible, with low toxicity and ease of clearance. They can be synthesized by designing their characteristics according to the foreseen administration path, or to the target of the transported drug. The analytical methods mostly used to evaluate the characteristics of lipid nanosytems for drug delivery involve studying their effects on cells, in vitro and in vivo. Although it is often considered a “niche technique“ for research in the bio-related sciences, Small Angle X-ray Scattering (SAXS) is a versatile tool to study the structure of nanosystems based on lipids, both ex situ and in situ. Therefore, it allows to evaluate both the effect of the different synthesis parameters and of the exposure of lipid nanoparticles to physiological conditions, which is of fundamental importance to design efficient drug delivery systems. In this mini-review, we will report some recent examples of characterization and design of nanoparticles based on lipids, where SAXS has been a fundamental step both to guide the synthesis of nanomaterials with tailored characteristics, and to understand the interaction between nanomaterials and cells.

## 1. Introduction

Interest in nanoparticles grew in the 1950–1960s, when the first studies on their potential applications for drug delivery started. The idea was to use the nanoparticles as sort of targeted bullets that could directly interact with the pathogens, and thus, improve the therapeutic effect of the administered drugs. On the other hand, nanomaterials could be used to incapsulate molecules or antigens and achieve a continuous, prolonged delivery to enhance the immune response, thus, making easier the administration of vaccines that usually required multiple injections [1]. Many molecules have been used since then to produce nanoparticles with therapeutic purposes: the first attempts included toxic substances such as acrylamide, and were then soon replaced by other compounds with better biocompatibility [2].

Since these pioneering works, in the following decades, further significative advances have made synthesizing biocompatible materials capable of delivering a number of therapeutic substances on site possible, allowing to target the release of drugs and to increase their bioavailability [3,4]. Nanoparticles for biomedicine are currently available in different formulations: depending on the chemical characteristics, they show special features which are convenient, for example, controlled drug release or selective interactions with the target. Ideally, nanoparticles with potential therapeutic use are non-toxic, non-immunogenic, stable during in vivo circulation and are able to deliver the encapsulated drug in the desired site [5]. Despite their huge potentiality, many nanomaterials still present problems in terms of toxicity, distribution and clearance in the body [6], e.g., in case of long-term administration, such as for treatment of chronic diseases [7].

The steps towards fully biocompatible and site-specific nanoparticles for medical applications are closely linked to the deep understanding of their interaction with biological targets, such as cells, biomembranes and proteins.

From this perspective, biopolymers are very attractive because of their affinity with cell membranes, low toxicity, ease of preparation and structural tunability that make them ideal materials for the design of controlled drug delivery systems [8,9,10]. Lipid nanoparticles, in particular, proved to be effective in enhancing the availability of even poorly soluble molecules due to their aptitude to promote the formation of solubilized phases during digestion [11,12]. In the 1970s, the potential of liposomes for therapeutic applications were started to be considered, but several years were necessary before this interest resulted in the first official approval for injectable formulation, in the early 1990s [13].

Later on, the synthetic technology improvements and new scientific evidences turned out in the design and production of lipid nanoparticles composed of solid matrix of nontoxic lipids or waxes with an averaged dimension of 100–300 nm, which were able to protect from degradation the incorporated drugs.

In the following years, the discovery of non-lamellar liquid crystalline nanoparticles—mainly hexosomes and cubosomes, which are suited for the loading of lipophilic molecules, and of lipid–drug conjugates, which improve the loading capacity of hydrophilic drugs—opened new exciting opportunities in the field of molecule delivery [14,15,16].

Cubosomes and hexosomes are, presently, widely studied for the production of stable, biocompatible, non-immunogenic drug delivery formulations. They are typically synthesized by coupling amphiphilic lipids such as Monooleyn (MO) or Phytantriol doped with a suitable stabilizing agent such as triblock copolymers—for example, Pluronic^®^ F127—or with natural compounds, such as casein, ethyl cellulose, modified starch or food-derived emulsifiers, such as citrem [17].

The crystal structure of lipidic nanoparticles seem to have an impact on the effectiveness of drug release [18]: since the number of molecules released by cubosomes is higher than hexosomes due to the smaller pores and surface area of the latter, this structural characteristic can be used to tune the drug release in response to different external stimuli, such as temperature or pH, which induce a phase transition in the lipid mesostructure [19].

A common strategy to understand the therapeutic potential of nanoparticles is the study of their interaction with living cells [20] to improve absorption and drug release once internalized. The cargo particles should be tailored to the specific therapeutic goal and on the administration route. A detailed characterization of the mesostructure of non-lamellar liquid crystal nanoparticles, as well as an in situ study of their response to environmental conditions in vitro and in vivo, is thus, essential.

In situ time-resolved chemical and structural characterization is of paramount importance for the design and synthesis of lipid-based nanomaterials with potential therapeutic applications. In this respect, many analytical techniques are available, which allow for determining the crystalline structure, the chemical composition and the stability against aggregation in solution, to name a few [21,22].

Commonly used methods for the morphological characterization of lipid nanoparticles include Dynamic Light Scattering (DLS), which allows for investigating the size distribution; Zeta potential, which gives information on the surface charge and the stability of nanoparticles in a solution; or electron microscopy techniques such as SEM and TEM, which are useful to gain insights on the overall shape and the structural features of the nanoparticles in a localized area. These techniques complement nicely the structural information that could be obtained with Small Angle X-ray Scattering (SAXS).

In scattering techniques, the whole illuminated volume of the sample is investigated, thus, average values of the structural parameters can be obtained, in contrast to microscopy, where a single object or a small part of the sample can be investigated. Moreover, SAXS allows for in situ and time-resolved studies to be carried out in a much simpler and faster way than TEM and SEM, allowing for changing the sample environment in terms of temperature, pressure, chemical composition, for example. DLS gives details on the average size and size distribution of particles in a sample, but does not inform on the shape of the particles.

SAXS is an analytical technique that takes advantage of the interaction of X-rays with the electrons of the analyte. Once treated with the appropriate mathematical model, the scattered radiation pattern collected at the detector gives information on the supramolecular envelope of the studied material [23], thus, on the size, shape and monodispersity of the nanoparticles in the present case. It also allows for investigating changes in the crystalline structure in response to external stimuli. SAXS was developed in the late 1930s, and since then, it has been widely used for the characterization of the structure and of the dynamics of nanosized materials [24]. In the last few decades, it has been profitably applied to structural biology because it offers the unprecedented possibility to elucidate the structure of macromolecules in a solution as well as to study the structural properties of biomembranes in an environment that mimics physiological conditions [25]. Thus, it is a powerful tool to investigate in situ both lipid nanoparticles structures and their behavior when exposed to biologically relevant environment. Small and Wide-Angle X-ray Scattering (SWAXS) data can be acquired simultaneously to investigate the intramolecular distances and interactions to evaluate the eventual crystalline structure of the loaded molecules [26], or to elucidate the perturbative effect of drugs on lipid model biomembranes [27].

Nowadays, excellent bench apparatuses for SAXS and WAXS analysis are available. On the other hand, the time resolution that is necessary to obtain a good signal-to-noise ratio with such instruments may be of minutes or even hours, depending on the electron density contrast of the analyte. Therefore, for time-resolved studies, e.g., to monitor the phase transition in response to a change in pH, synchrotron SAXS is the technique of choice because it requires just a few seconds of exposure, thus, increasing the efficiency of the measurements while limiting radiation damage on the exposed sample.

SAXS analysis is often coupled with SANS (Small Angle Neutron Scattering) for the structural and composition study of nanoparticles [28] and biomembranes [29]. Analyzing the sample with both techniques allows for refining the structural model of lipid nanoparticles [30]. While X-rays interact with electrons, neutrons interact with the nuclei of atoms: neutron radiation is non-destructive, thus, avoiding any radiation damage issue. The contrast variation method in SANS allows for determining the distribution of the components in complex nanoparticles, such as different lipids, excipient, stabilizers and even loaded molecules, e.g., mRNA in the recently developed COVID-19 vaccines [31,32,33].

In the present review, we will focus on synchrotron Small Angle X-ray Scattering (SAXS) experiments aimed to determine the best structure and morphology of lipid-based nanoparticles for therapeutics. We will report some case studies in which SAXS was of pivotal importance to optimize the efficiency of lipid nanoparticles as cargo systems, as well as to design new formulations with improved stability or tailored on the administration route.

Table 1 summarizes the systems described in this review grouped by their foreseen applications, and the objective of the SAXS investigations performed therein.

## 2. Phase Transition of Lipid Nanoparticles for Controlled Drug Delivery

Liposomes, cubosomes and hexosomes allow for incorporating lipophilic and hydrophilic drugs: their formulations can be stabilized with additives that increase the embedded drug stability and offer the possibility to trigger the release of the substances, making them ideal vectors for target drug delivery systems [50,51].

Adjusting the nanoparticle composition allows for creating drug delivery tools with specific responsive characteristics that can be investigated in situ with SAXS. With this information, lipid nanoparticles for therapeutic use can be designed according to the administration route, or to the target site.

It was recently demonstrated in an experiment in vivo that cubosomes obtained by coupling selachil alcohol with Tween^®^ 80 as a stabilizer were more effective in delivering the poorly water soluble drug Phenytoin to the brain, with respect to hexosomes derived from the same lipid, but using Pluronic^®^ F127 as stabilizing agent [52]. This better performance was attributed to the increased negative curvature of the lipid bilayer induced by Phenytoin in the presence of Tween80, which is more internalized in the lipid bilayer with respect to Pluronic F127. Usually, the lipid composition must be adjusted in order to change the lipid curvature to induce a phase transition. In the reported study, simple formulations composed of one single lipid were produced, and the phase-change was obtained by tuning the stabilizer.

Lipidic nanoparticles are particularly attractive for oral drug administration [53]. They protect the loaded drug from degradation in the gastrointestinal tract increasing the circulation time, and can be coupled with natural compounds, such as natural oils or fatty acids, to enhance pH responsiveness. Lipidic drug carriers must resist the very acidic stomach environment, and dissolve to release the loaded formulation once in contact with the neutral blood pH.

Understanding the dissolution mechanism is a critical aspect of delivery systems design: in this respect, SAXS offers the possibility to study in situ the lipolysis during digestion. Simultaneous Small and Wide-Angle X-ray Scattering (SWAXS) allows for thoroughly evaluating the extent of drug dissolution, as the changes in crystalline structure of the internalized drugs can also be observed [54]. The formation of novel structures during lipolysis—thus, during the digestion of the orally administered formulation—can affect the solubility of the nanoparticles, reducing the drug absorption. As recently reported, drug carriers based on natural surfactants have great potential for the production of Self Emulsifying Drug Delivery Systems (SEDDS). It was recently demonstrated in a study on coupled in situ SAXS and ex situ Cryo-TEM, that during in vitro lipolysis, SEDDS undergo a structural evolution that is correlated with the amount of the surfactant in the formulation [39].

**Figure 1:**
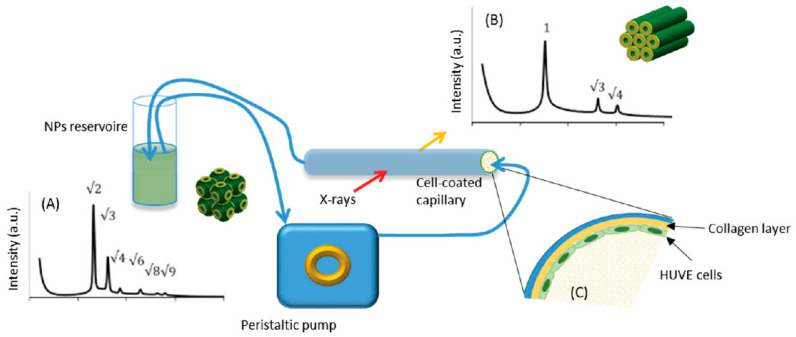
Scheme of the experimental setup. Phase transition from (**A**) cubosomes to (**B**) hexosomes, (the respective Bragg peaks indexing are indicated), when in contact with a glass tube coated with human umbilical vein endothelium cells (HUVE), was observed in situ with SAXS. (**C**) Detail of the observation cell coated with human umbilical vein endotheliumcells. (Adapted from [41]).

Elucidating the fate of nanostructured lipid nanoparticles when they come into contact with target cells is of paramount importance. Recently, the phase-change of non-lamellar lipid nanoparticles when they interact with cells was studied in situ with SAXS. A 3D model simulating the endothelium of human umbilical vein (HUVE) was developed, and the evolution of cubosomes to hexosomes during circulation was investigated [41]. Umbilical cells were seeded on the inner surface of a glass capillary, and the cubosomes’ dispersion was subsequently flown through it (Figure 1). The flow-through cell was exposed to X-rays, and the phase transition of cubosomes into hexosomes during contact with the cells was observed. These experiments demonstrated the potentiality of a structural study in real time.

### 2.1. External Stimuli Responsive Lipid Nanoparticles

To design drug delivery nanoparticles that are responsive to external stimuli, understanding how to tune the phase transition is a critical aspect. It is usually induced by a change in temperature or in the pH of the medium, and it can be exploited to determine the drug-release extent.

**Figure 2:**
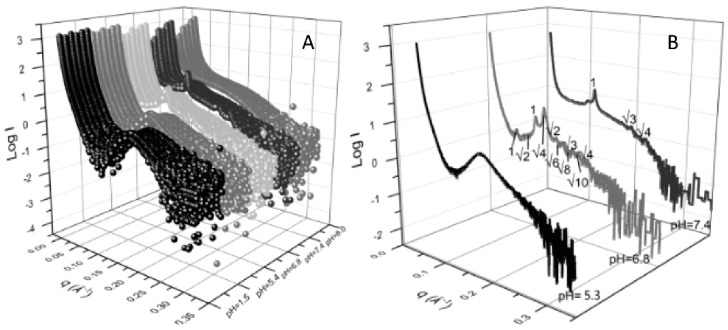
(**A**) SAXS patterns obtained from nanoparticles of different composition (front to back: MO:OA, MO:0.9OA:0.1BJO, MO:0.8OA:0.2BJO, MO:0.7OA:0.3BJO, MO:0.6OA:0.4BJO and MO:0.5OA:0.5BJO) collected at different pH (pH = 1.5, 5.3, 6.8, 7.4, and 8.0, front to back); (**B**) SAXS pattern of lipid nanoparticles loaded with oleic acid and Brucea javanica oil (MO:0.9OA:0.1BJO) collected at pH 7.4 (healthy cells), 6.8 (tumor cells proximity) and 5.4 (tumor cells endosomes). The Bragg peaks indexing of inverted hexagonal (pH 7.4) and inverted cubic (pH 6.8) liquid crystalline phases is indicated (Reproduced with permission from [34]).

By loading lipid nanoparticles composed of MO and oleic acid (OA) with the antitumor drug Doxorubicin and with a natural oil obtained from *Brucea javanica* (BJO)—a traditional medical herb with antiproliferative properties on cancer cells—Li and collaborators obtained pH-sensitive lipid nanoparticles capable of converting from hexagonal to cubic crystalline phase to emulsified microemulsions, in response to the lower pH in proximity and inside tumor cells. SAXS analysis demonstrated the pH responsiveness and the effect of Brucea Javanica oil on the nanoparticles’ stability (Figure 2). At pH 7.4, hexosomes with an internal HII-phase structure were formed: at a lower pH (6.8), the crystalline structure changed to cubosomes, with an inner structure where two phases (Pn3m and Im3m) coexist [34].

**Figure 3:**
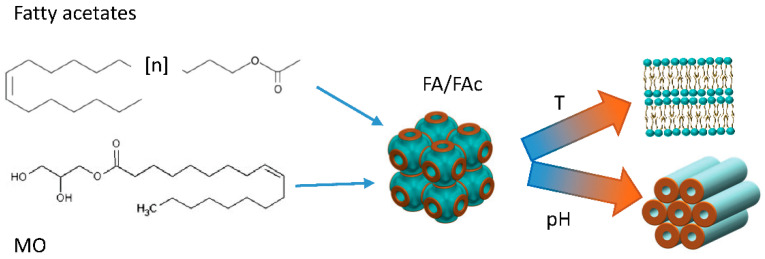
Cartoon representing the experiment reported in [35]. The phase transition from cubosomes to hexosomes of MO nanoparticles produced with different amounts of Fac and FA in different molar ratios can be controlled with temperature and pH variations.

By modifying their composition, it is possible to tailor the response to the external conditions and to create a “library” of pH- and temperature-responsive nanoparticles, whose crystalline structure can be tuned for fast (cubic) or slow (hexagonal) drug release. Recently, lyotropic liquid crystals with cubic Fd3M symmetry obtained from MO were doped with various types and amounts of fatty acids (FA) and fatty acetates (FAc) with different chain lengths (Figure 3). By adjusting the nanoparticles composition, it was possible to produce a collection of hybrid MO-FA–FAc micellar nanoparticles which exhibited a Fd3m structure. As demonstrated by in situ SAXS, these cubosomes underwent cubic to lamellar and cubic to hexagonal phase transitions in response to temperature and pH changes, respectively, depending on the chain length, the amount and the respective molar ratio of FA and FAc incorporated in the formulation [35].

In another experiment, a set of synthetic ionizable aminolipids was designed and used to dope MO nanoparticles stabilized with the surfactant Pluronic^®^ F-127. SAXS allowed for investigating the phase transition of MO in response to different pH of the several formulations obtained by adjusting the nanoparticles’ composition [36].

### 2.2. Hybrid Lipid-Based Nanoparticles

Lipids can be associated with other biocompatible molecules, such as polysaccharides, in order to induce the formation of structured mesophases with high stability and specificity for applications where bioadhesion is desired. Coupling MO with Pluronic^®^ F127 as a stabilizer, chitosan-N-arginine and alginate lead to the formation of pH responsive cubosome nanoparticles. SAXS experiments allowed for determining the proper amount of stabilizer needed to obtain nanoparticles characterized by cubic symmetry, to gain insight in the phase transition in response to pH change as well as on the distribution of chitosan-N-arginine inside the lipid structure [37].

**Figure 4:**
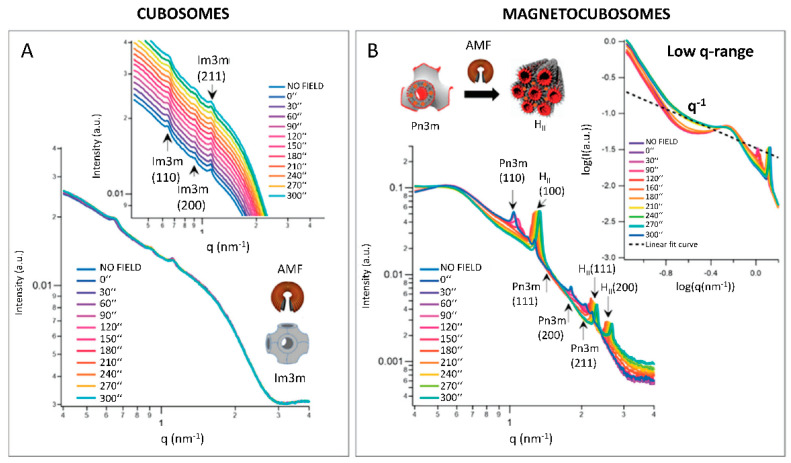
Response of (**A**) GMO cubosomes and (**B**) superparamagnetic cubosomes (showing a phase transition from Pn3m (cubic) to HII (hexagonal) crystalline phase) exposed to an alternated magnetic field. The Miller Indexes for the respective crystalline phases are indicated. Each SAXS profile corresponds to a different application time of the magnetic field. Inset in (**A**) highlights the scattering peaks of cubosomes. Inset in (**B**) represents a magnification of the low-q region of the scattering profile. The pearl-necklace-like arrangement of magnetocubosomes after 210 s of exposure to the magnetic field is evidenced by the appearance of a q^−1^ scalar law. (Reproduced with permission from [42]).

Lipids can be also coupled with inorganic materials, with a great potential for localized drug release. Caselli and collaborators studied the behavior of hybrid paramagnetic nanoparticles when exposed to a magnetic field: they exploited the capability of lipidic-ordered liquid crystals to change the phase from cubic to hexagonal, to trigger the localized drug release. The phase-change was accompanied by a massive water ejection that could promote the release of hydrophilic drugs embedded in the lipid nanoparticles. By coupling superparamagnetic iron oxide nanoparticles with 1-monoolein (GMO) nanoparticles, they produced hybrid cubosomes with Pn3m cubic symmetry, which changed to hexagonal in response to the heat generated by the magnetic nanoparticles exposed to a low-frequency alternating magnetic field [42]. With SAXS, it was possible to characterize in situ the lipid phase transition either during the exposure to temperature only, or to an alternating magnetic field. In the absence of a magnetic field, the phase transition from Pn3m to HII was observed at temperatures compatible with physiological conditions (below 40 °C) [55]. Both pure GMO cubosomes and hybrid nanoparticles were exposed to the alternating magnetic field: SAXS data confirmed the thermotropic effect of the superparamagnetic iron oxide on the crystalline structure of the hybrid cubosomes once exposed to the magnetic field, while no phase-change was observed in the pure cubosomes. SAXS data analysis also revealed a pearl-necklace-like arrangement of the hybrid magnetic cubosomes after 210 s of exposure to the magnetic field (Figure 4).

## 3. Functionalization with Polyethylene Glycol (PEG)

Functionalization of lipid nanoparticles with PEG has several advantages in the design of drug delivery systems [56]: PEG is commonly used to coat nanoparticles aiming to prevent aggregation and avoid phagocytosis, thus, increasing their residence time in the circulating blood; it also interferes in the formation of molecular corona on the nanoparticles [43]. PEG can be incorporated in the formulation as a pure component, or covalently linked to one of the lipids present in the formulation (PEGylated lipids). PEG creates a hydrophilic shell on the surface of lipid nanoparticles, which inhibits the desorption of drugs, particularly hydrophobic molecules that are encapsulated in the nanoparticle core. PEG molecules grafted on the surface also allow for accommodating a larger amount of hydrophilic drugs into core–shell nanoparticles, because they do not occupy space inside the nanoparticle [57].

Liposomes are promising tools for the delivery of antitumoral drugs. In order to reach the therapeutic target, they must resist the extracellular matrix (ECM). Aiming to mimic such an environment, Bandara and coworkers studied the effect of exposure to hydrogels composed of gelatin, alginate, PEG and the commercial ECM Matrigel^®^ on DOPC (1,2-dioleoyl-sn-glycero-3-phosphocholine) liposomes. The structural features of the liposomes before and after exposure to the hydrogel were studied with SAXS. Their results showed that once exposed to the extracellular matrix, DOPC liposomes turn to multilamellar vesicles, and form aggregates of micrometer size. Adding a small amount of PEGylated DOPE (1,2-Dioleoyl-sn-glycero-3-phosphoethanolamine) to the liposome formulation, the formation of a PEG corona reduced the extent of liposomes aggregation, which has important implications for stability and immune responses [58].

In a systematic high-throughput study conducted by Sarode et al., it was demonstrated that the PEG-to-lipid molar ratio and length of the PEG carbon tail have significant effects on the particle size and on the loaded molecule delivery. SAXS data demonstrated that when a shorter-chained, negatively charged PEGylated lipid is used, the crystalline structure of the resulting nanoparticle is more ordered, with respect to longer-chained PEGylated lipids with a neutral charge, resulting in less-effective drug delivery efficiency [59].

## 4. Coated Nanoparticles

### 4.1. Biomolecular Corona

In vivo, lipid vectors for drug delivery adsorb proteins, lipids and other plasmatic biomolecules on their surface in a non-specific manner once injected: depending on the nanoparticles’ properties, such as their charge, hydrophilicity, dimensions and morphology, the several components present in plasma interact and build a coating. The resulting biomolecular corona might interfere with the nanoparticle–cell interaction, induce an immune response and alter the drug-release efficiency [60].

Liposomes commonly used as vectors for therapeutic molecule delivery can be unilamellar vesicles, where the hydrophilic drug molecules are loaded in the central aqueous core of the nanoparticle, or multilamellar vesicles, which can host lipophilic drugs within the lamellar structure. The majority of liposomes commercially available for drug delivery purposes are Small Unilamellar Vesicles (SUV) [61]—although they are stable during circulation, the interaction of proteins with the SUV surface provokes the formation of multilamellar structures and of liposome clusters: the change in the crystalline structure implies the rupture of the vesicles, which may result in the release of the loaded drug.

The formation of larger aggregates may impede the cellular uptake of the particles. The latter effect, however, becomes less evident as the concentration of proteins attached to the nanoparticles surface increases, possibly due to the repulsive interaction of charged proteins, as demonstrated by Caracciolo and coworkers [44]. In a recent experiment, they used a commercial formulation of liposomal Doxorubicin, a drug used for cancer treatment, to study the effect of biomolecular corona on nanoparticle stability and drug release, incubating the liposome–drug formulation in the concentration of human plasma that mimics the physiological conditions. TEM, DLS and Zeta potential experiments demonstrated the formation of biomolecular corona already at a low plasma concentration, which led to an increased particle size and to a change in the particle surface charge. SAXS characterization of the drug-loaded nanoparticles before and after incubation in human plasma demonstrated the absence of the multilamellar structure, showing that the presence of the biomolecular corona did not lead to the formation of a multilamellar structure; moreover, a fluorescence-lifetime imaging microscopy analysis on the drug-loaded liposomes after incubation revealed that the amount of Doxorubicin in the incubated particles was significantly lower with respect to the pristine ones, suggesting that the exposure to human plasma might degrade the liposomes, altering their persistence in the blood circulation.

On the other hand, protein coating on liposome carriers (namely, protein corona), is effective in protecting the nanoparticles from degradation in blood, reducing the interaction with leukocytes and prolonging the circulation time in vivo. Giulimondi and colleagues recently demonstrated the protective effect against uptake by blood macrophages of pre-adsorbed human plasma (HP) protein corona on liposomes obtained from cationic, neutral and anionic lipids. SAXS experiments allowed for evaluating the different effects on the mesostructure of increasing concentrations of plasma proteins adsorbed on the surface of liposomes—these observations demonstrated that the nanostructure of liposomes pre-coated by HP protein corona depends on the surface charge of the uncoated liposomes. Following these results, the authors showed that the protein corona of pre-coated cationic liposomes is not affected by the interaction with human plasma, suggesting that the nanoparticles’ ability to evade sequestration by leukocytes during circulation remains stable. The authors demonstrated that at a low concentration, human plasma protein corona prevents the internalization of cationic liposomes into macrophages, but does not have the same effect on neutrally charged or anionic liposomes. Their findings suggest the possibility to use drug-loaded cationic liposomes coated with low-concentration protein corona to prolong blood circulation and facilitate drug delivery to the target sites [40].

### 4.2. Others

Lipid nanoparticles can be coated with substances that enhance their adhesion properties to cells. Moreover, they may be used as vectors to facilitate the transport of smaller nanoparticles into the cells. Recently, chitosan-N-acetylcysteine-coated unilamellar liposomes were reported to improve the bioavailability of Seleniun nanoparticles in the small intestine. The availability of –SH groups on the coated liposomes’ surface is critical for their adhesion to the intestine cells; in fact, -SH groups are possibly interacting with mucine glycoproteins via a disulfide bridge formation. As demonstrated by in vivo observations, uncoated particles did not adhere to the cells surface, confirming the efficiency of the chitosan coating. In the reported study, SAXS evidenced that the chitosan coating did not affect the particles integrity, which is necessary to guarantee the interaction with the cells surface and subsequent diffusion of Selenium nanoparticles into the intestinal mucosa [38].

## 5. Encapsulation Strategies for Gene Delivery

The therapeutic use of nucleic acids is a tricky process because they are very sensitive and can easily degrade during circulation in the blood. Encapsulation of nucleic acids into nanoparticles increases their circulation time.

Lipid-based systems designed for gene delivery such as lipoplexes, which are composed of RNA/DNA and cationic lipids, are promising tools for gene delivery into cells [47].

Understanding the structure of the loaded compound with respect to the cargo particle is particularly important when the molecules to be delivered are nucleic acids, such as in the case of the new therapies based on mRNA [45,62,63].

**Figure 5:**
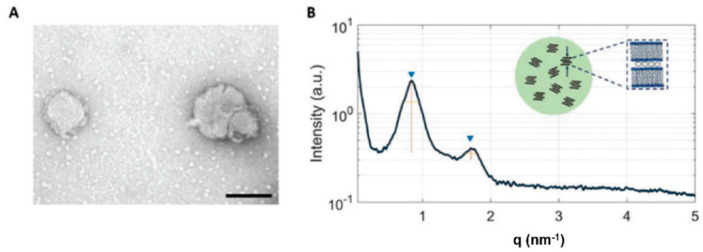
(**A**) TEM image of PEGylated nanoparticles (scale bar = 100 nm). (**B**) Scattering pattern of PEG-ylated nanoparticles. Inset: cartoon describing the nanostructure with multiple randomly oriented scattering domains. The broad peaks (▼) show the lamellar periodicity along the normal to the lipid bilayer, which includes the contribution of the membrane thickness and of the thickness of the water/DNA layer. (Reproduced with permission from Ref. [48]).

The traditional synthetic method to produce lipid–nucleic acid complexes is the self-assembly of lipid unilamellar vesicles obtained by the hydration of lipid films, and the subsequent passive diffusion of nucleic acid into them. This leads to an uncontrolled interaction between nucleic acids and lipids, and to poor loading. The microfluidics technique was recently used to improve the encapsulation efficiency of nucleic acids [48] Lipolexes were produced by injecting a lipid–PEG mixture and DNA plasmid into a commercial microfluidic device. The physio-chemical characterization of the obtained nanoparticles with SAXS showed the expected lamellar stacking of lipid layers as well as a different arrangement of the encapsulated DNA, with respect to that obtained by passive diffusion. In fact, in microfluidic-produced lipoplexes, DNA is not densely packed inside the lipoplexes. Loosely packed DNA may interact more easily with the cellular membrane and the release into the cell might be facilitated (Figure 5).

The administration route of nanoparticles for therapeutics is a challenge, especially when the therapy is directed against chronic diseases that require long administration times. In these cases, it is of pivotal importance to promote the delivery of the loaded drug to the site of disease, minimizing the interaction with other body districts and enhancing the internalization in the target cells. A recent study by Conte and collaborators [49] demonstrated the possibility to deliver siRNA to the lung via an aerosol route, overcoming the mucus barrier that may trap the inhaled nanoparticles and neutralize the therapeutic siRNA absorption. To reach this goal, revealing the interaction between the cells and the siRNA carrier particle composition was essential. They synthesized hybrid nanoparticles (hNPs) were composed of a core of poly(lactic-co-glycolic) acid (PLGA) and a lipid shell to encapsulate a pool of siRNA directed against the nuclear factor involved in severe acute and chronic inflammation, as in the case of patients affected by cystic fibrosis. They used SAXS to confirm the core–shell structure of the synthesized particles as well as their finite size and monodispersity. SAXS data analysis also allowed for determining the thickness of the lipid shell layer, which proved to be essential in promoting the internalization of nanoparticles into the cells. SAXS structural analysis of hNPs in contact with cellular mucus showed the presence of slightly larger nanoparticles, which were attributed by the authors to a rearrangement of the mucus network around the nanoparticles. Nevertheless, this does not affect the efficiency in terms of siRNA release in the cells, as demonstrated by in vitro experiments on Calu3, a model cell line for cystic fibrosis.

Polysarcosine is a synthetic compound based on repetitive units of the amino acid sarcosine, which can be used to functionalize the surface of lipidic nanoparticles. It has stealth-like properties similar to those of PEG, but polysarcosine-coated nanoparticles show a lower clearance in the blood with respect to PEG-ylated ones, and do not induce an immune response or hypersensitivity in patients [64,65]. Moreover, these nanoparticles show higher transfection efficiency if compared to PEG-ylated nanoparticles, which is desirable for the design of nanoparticles for mRNA delivery.

Recently, mRNA-loaded nanoparticles coated with polysarcosine were synthesized and tested as gene delivery tools in transfection experiments on hepatocarcinoma cell lines, as well as in in vivo experiments. SAXS analysis revealed information on the surface structure as well as on the internal organization of the nanoparticles and loaded RNA, which are important parameters to design nanoparticles tailored on the specific therapeutic application [46].

## 6. Outlook and Future Perspectives

The great push for research on the synthesis and optimization of lipid-based vectors for drug delivery that has taken place in recent years, also following the COVID-19 pandemic, has further shed a bright light on the potential of these nanomaterials for therapeutic or preventive applications. The environmental responses as well as the particle–cell interaction characteristics of the formulations proved the importance of properties such as dimension, structure and dosage of functional materials designed for therapeutic applications. In this perspective, synchrotron SAXS, with its in situ and time-resolved capabilities, has already proven to be an important technique to gain structural information, which is fundamental for drug delivery design and development.

Further studies are necessary to develop drug-loaded lipid nanoparticles and hybrid nanocomposites that will be able to maintain their integrity during circulation. In this respect, both in situ and ex situ SAXS are particularly suitable techniques.

Future challenges to be addressed in the near future include:

- The design of compounds able to improve the targeted release of drugs exploiting the properties of the molecular corona. The aim is to promote its interaction with specific cell receptors;
- The tailoring of lipid nanoparticles capable of reaching the disease sites and produce therapeutic activity selectively at the target organ;
- The achievement of an effective triggered drug release via the exposure to light, pH variation and temperature.

All these ambitious goals rely directly on the physio-chemical characteristics of the NPs—understanding the interaction with model membranes in situ, or by means of models reproducing the target organs or tissues, will help to achieve better functionality and performance.

## Figures and Tables

**Table 1:** Summary of the experiments described in the text, grouped by the foreseen application.

Application	System	In Situ/Ex Situ SAXS	Investigation on	Ref.	Outcome
pH-dependent drug release	Cubosomes (MO + Oleic acid + natural derived oil)	Ex situ formulation design	Stability and pH responsivity	[34]	Optimized formulation
	cubosomes(MO + fatty acetates)	In situ pH responsivity	pH and temperature responsivity	[35]	Improved formulation for pH-dependent phase change
	Cubosomes (MO + F127 + aminolipids)	In situ Investigate structural changes at different pH	pH responsivity	[36]	Improved formulation–pH-dependent phase change
Oral drug delivery	cubosomes (Mo + F127 + polysaccharides)	Ex situ Formulation design	pH responsivity	[37]	Improved formulation–pH-dependent phase change
	Liposomes (chitosan-N-acetylcysteine)	Ex situ Effect of coating on NPs stability	Improvement on the interaction with intestine cells	[38]	Lipid bilayer integrity confirmed after polymer coating
	Self-emulsifying drug delivery systems	In situ structural changes during digestion	Poorly soluble drug cargo stability during lipolysis	[39]	Selection of excipient to improve SEDDS stability
Enable prolonged circulation in vivo	Liposomes/protein corona	Ex situ “Tailor-made” protein coating of NPs	Stability during circulation	[40]	Plasma protein coating induce structural changes in liposomes
Interaction betw.lipid-based NPs and live cells	cubosomes (Phytantriol–F127)	In situ Phase transition in contact with HUVE cells	Interaction between structured NPs and cells	[41]	Understanding the dynamics of vascular endothelial cells–lipid NPs interactions
Thermally triggered drug/nutrients delivery	cubosomes (MO + superparamagnetic iron oxide)	In situ Investigate phase transition	Temperature responsivity and magnetic trigger	[42]	Formulation of responsive magnetocubosomes
Active prostatic cancer cell targeting	PEG-ylated liposomes	Ex situ Effect of PEG chain length on protein corona	Stability, reduction in immune response	[43]	Formulation improvement of PEGylated cationic lipid NPs
Delivery of Doxorubicine for cancer treatment	Liposomes/biomolecular corona	Ex situ Stability after plasma incubation	Drug delivery efficiency	[44]	Commercial formulation stability assessment
mRNA transfection	Lipoplexes (DOTAP/RNA/Protamine)	Ex situ Formulation design	Physico-chemical particles characterization	[45]	Formulation improvement for better transfection efficiency
	Lipoplexes/Polysarcosin	Ex situ Particles design	Improvement of stability	[46]	Optimized formulation
DNA transfection	Lipoplexes (Cationic lipids/Cholesterol/DNA)	Ex situ Formulation design	Physico-chemical characterization	[47]	Optimized formulation
	Lipoplexes (Cationic lipid/DNA/PEG)	Ex situ Formulation design	Microfluidics for NPs synthesis	[48]	Optimized pDNA loaded NPs production
siRNA delivery	lipid shell/PLGA core/RNA	Ex situ Stability in physiological conditions	Design of aerosol drug delivery systems	[49]	Understanding the dynamics of lung cells–lipid NPs interactions

## Data Availability

Not applicable.
